# Expression microarray identifies the unliganded glucocorticoid receptor as a regulator of gene expression in mammary epithelial cells

**DOI:** 10.1186/1471-2407-14-275

**Published:** 2014-04-22

**Authors:** Heather D Ritter, Christopher R Mueller

**Affiliations:** 1 Queen’s Cancer Research Institute, Queen’s University, Kingston, Ontario, Canada K7L 3N6; 2 Department of Biomedical and Molecular Sciences, Queen’s University, Kingston, Ontario, Canada K7L 3N6; 3 Department of Pathology and Molecular Medicine, Queen’s University, Kingston, Ontario, Canada K7L 3N6

**Keywords:** Glucocorticoid receptor, Unliganded, Hydrocortisone, Expression microarray, Breast cancer, BRCA1

## Abstract

**Background:**

While glucocorticoids and the liganded glucocorticoid receptor (GR) have a well-established role in the maintenance of differentiation and suppression of apoptosis in breast tissue, the involvement of unliganded GR in cellular processes is less clear. Our previous studies implicated unliganded GR as a positive regulator of the *BRCA1* tumour suppressor gene in the absence of glucocorticoid hormone, which suggested it could play a similar role in the regulation of other genes.

**Methods:**

An shRNA vector directed against GR was used to create mouse mammary cell lines with depleted endogenous levels of this receptor in order to further characterize the role of GR in breast cells. An expression microarray screen for targets of unliganded GR was performed using our GR-depleted cell lines maintained in the absence of glucocorticoids. Candidate genes positively regulated by unliganded GR were identified, classified by Gene Ontology and Ingenuity Pathway Analysis, and validated using quantitative real-time reverse transcriptase PCR. Chromatin immunoprecipitation and dual luciferase expression assays were conducted to further investigate the mechanism through which unliganded GR regulates these genes.

**Results:**

Expression microarray analysis revealed 260 targets negatively regulated and 343 targets positively regulated by unliganded GR. A number of the positively regulated targets were involved in pro-apoptotic networks, possibly opposing the activity of liganded GR targets. Validation and further analysis of five candidates from the microarray indicated that two of these, *Hsd11b1* and *Ch25h*, were regulated by unliganded GR in a manner similar to *Brca1* during glucocorticoid treatment. Furthermore, GR was shown to interact directly with and upregulate the *Ch25h* promoter in the absence, but not the presence, of hydrocortisone (HC), confirming our previously described model of gene regulation by unliganded GR.

**Conclusion:**

This work presents the first identification of targets of unliganded GR. We propose that the balance between targets of liganded and unliganded GR signaling is responsible for controlling differentiation and apoptosis, respectively, and suggest that gene regulation by unliganded GR may represent a mechanism for reducing the risk of breast tumourigenesis by the elimination of abnormal cells.

## Background

Hormonal signaling plays an integral role in the regulation of mammary gland function and differentiation. *In vivo*, the glucocorticoid hormone cortisol is involved in the maintenance of breast functional differentiation during the latter stages of pregnancy, where it induces the formation of the rough endoplasmic reticulum [1], and regulates the release of milk proteins [2]. Following weaning, a decrease in circulating levels of cortisol is responsible for the onset of the apoptotic process of involution, where the mammary tissue morphology is reverted to a quiescent state [3]. The nature of cortisol’s ability to suppress apoptosis in the breast appears to be dependent on the cellular differentiation state, since glucocorticoids induce cell cycle inhibitors such as p21 in undifferentiated cells, while they reduce their expression and inhibit apoptosis in differentiated cells [4]. The intracellular receptor for cortisol, the glucocorticoid receptor (GR), is ubiquitously expressed in the human breast, being observed in the nuclei and cytoplasm of both luminal epithelial cells and myoepithelial cells, as well as in the nuclei of stromal cells, endothelial cells, and adipocytes [5-7]. GR-knockout mice die shortly after birth due to lung immaturity and respiratory failure, illustrating that expression of GR is essential for life [8]. Consequently, mutagenesis and Cre-LoxP recombination targeting of breast epithelial cells in adult mice have been used to explore the role of GR in mammary gland development and function [1,9-11]. GR with a point mutation in the second zinc finger of the DNA-binding domain (exon 4; A458T) cannot bind a canonical Glucocorticoid Response Element (GRE), but retains its ability to transrepress gene expression through protein-protein interactions [9]. Virgin mice expressing this DNA-binding GR mutant exhibit impaired ductal development while lactating mice exhibit normally differentiated mammary glands capable of milk production, emphasizing that transcriptional regulation by protein-protein interactions, rather than DNA-binding, forms the basis of glucocorticoid action during this process [1]. In support of this, loss of breast epithelial GR results in delayed development of the lobuloalveolar compartment during pregnancy as a result of decreased cell proliferation, but during lactation, GR-deficient mammary epithelium is capable of milk production and secretion following increased epithelial proliferation after parturition in the mutant glands [10]. GR contributes to mammary lobular unit spatial formation through its ability to stimulate the expression of proteins essential for the spatial organization of the acini, such as the integrin beta-4 subunit [12]. It is clear that glucocorticoids and therefore liganded GR are essential for the growth and differentiation of the mammary gland, as well as the suppression of apoptosis; however, the role of unliganded GR in these processes has not been investigated.

Our previous studies indicated that unliganded GR is recruited to and positively regulates the *BRCA1* promoter through its interaction with the beta subunit of GABP. The addition of hydrocortisone (HC) abolishes this effect and results in decreased *BRCA1* expression [13]. The positive regulatory effect of unliganded GR appeared to be constitutive, involving basal GR levels within breast cells, since no stimulus or secondary messenger was required for its activation, unlike other reports of ligand-independent activation by other steroid hormone receptors which have typically been in response to other stimuli [14]. Consequently, our model of *BRCA1* activation by unliganded GR is a novel mechanism of GR regulation, and it is possible that the unliganded receptor may be involved in the regulation of multiple genes in this manner. Previous efforts to identify targets of GR regulation have involved expression microarray following treatment of human breast cells with dexamethasone, thus revealing genes both positively and negatively regulated by liganded GR (i.e. glucocorticoid-regulated genes) [15]. ChIP-chip analysis was used to investigate promoter occupancy by liganded GR and revealed that GR was bound predominately near genes responsive to glucocorticoids in A549 lung cells and not at genes regulated by GR in other cell types examined [16]. ChIP-seq analysis of GR binding sites in A549 cells revealed approximately 2600 genes that are weakly bound by unliganded GR [17], and although the identities of these genes were not investigated, this study suggested to us that gene regulation by unliganded GR is not only plausible but it may be widespread.

In the current study, we used an shRNA directed against GR to create mouse mammary epithelial cell lines with depleted endogenous GR expression. These cell lines were used to identify genes up and downregulated in the absence of endogenous unliganded GR expression using expression microarray. We found that in cells depleted of GR, 260 genes were significantly upregulated, while 343 genes were significantly downregulated. Since the downregulated genes represented those which are positively regulated by unliganded GR, potentially through a mechanism similar to that reported for *BRCA1*[13], we examined the most significant networks comprised of this gene set via pathway analyses, and determined that several of these genes were involved in pro-apoptotic networks. Validation and further analysis of five candidates of positive regulation by unliganded GR indicated that two of these, *Hsd11b1* and *Ch25h*, were also downregulated following HC treatment, in a manner similar to *Brca1*. Furthermore, GR was shown to interact directly with and upregulate the expression of the *Ch25h* promoter in the absence, but not the presence, of HC, confirming our previously described model of gene regulation by unliganded GR.

## Methods

### Cell culture and treatments

The non-malignant murine mammary epithelial cell line EPH-4, which was derived from spontaneously immortalized mouse mammary gland epithelial cells [18], was a gift of Dr. Calvin Roskelley (University of British Columbia, Vancouver, Canada). EPH-4 cells were cultured as previously described [13,19]. EPH-4 cells stably transfected with H1-2 empty vector or shGR (see below) were maintained in serum-free media with 2 μg/mL puromycin (Sigma). Cell treatments were completed using media lacking serum and containing either 1 μg/mL hydrocortisone (HC) (Sigma), 10 μM RU-486 (Sigma), or ethanol vehicle for 48 hours.

### DNA constructs

Creation of the L6-pRL *BRCA1* promoter construct, the H1-2 and shGR vectors, as well as GR FL and GRΔLBD (originally named GR TAD-DBD-HR) has been described previously [13,20]. The rat construct GRwt (wild-type GR) was a gift of Keith Yamamoto (University of California, San Francisco, USA), and its construction has been described previously [21]. The pCAGGS-GABPα and pCAGGS-GABPβ constructs were obtained from Hiroshi Handa [22]. The *Ch25h* promoter fragments Ch25h-9, Ch25h-10, Ch25h-11, Ch25h-11.5, Ch25h-12 were PCR amplified from EPH-4 genomic DNA using primers listed in Additional file 1: Table S1. To construct the *Ch25h* promoter reporter vectors, *Ch25h* PCR products were cut with Bam HI/Sal I and ligated into pRL-null (Promega), which was cut with Bgl II and Sal I. Each *Ch25h* promoter fragment was cloned into pRL-null upstream of the *Renilla* luciferase (R-luc) sequence.

### Transient transfections and luciferase assays

Approximately 24 hours prior to transfection, EPH-4, EPH-4 EV-50, or EPH-4 shGR-19 cells were plated in serum-containing medium on 12-well culture dishes at a density of 5 × 10^4^ cells/mL. Cells were transfected in triplicate with 1 μL per well of FuGENE®6 transfection reagent (Roche Applied Science). Control cytomegalovirus (CMV)-luc vector (Promega) was used at 25 ng per well, as were expression vectors and empty vector controls. The remainder of the 250 ng per well was allotted to the appropriate *Renilla* luciferase reporter vector. Cells were treated with HC or ethanol vehicle (as described above) in serum-free medium 24 hours following transfection. Forty-eight hours after treatment, cells were harvested for the Dual-Luciferase® Reporter Assay (Promega) as previously described [13,23].

### Creation of EPH-4 shGR stable cells

Approximately 24 hours prior to transfection, EPH-4 cells were plated in serum-containing medium on 100 mm culture dishes at a density of 5 × 10^4^ cells/mL. Cells were transfected with 11.25 μL per plate of FuGENE®6 transfection reagent along with 380 ng of pBABE-puro selectable marker and 3420 ng of either H1-2 empty vector or shGR (1:10 ratio). Following a 24 hour incubation, cells were lifted, diluted 1:20 and re-plated, and subsequently put into 2 μg/mL puromycin selection following another 24 hours. Colonies were lifted using filter paper, expanded, and cell lysates were screened by Western blot for GR protein levels using TBP as a loading control. The resultant stable cell lines EV-50, shGR-73, and shGR-19 were maintained with 2 μg/mL puromycin in media without serum.

### Western blot

Lysates were prepared in 1X SDS loading buffer and analyzed by standard Western blotting procedures. Polyvinylidene fluoride membranes (Millipore) were probed with the appropriate primary antibody: anti-GR (1:500; ab3579; Abcam), or anti-TBP (1:2,000; ab818; Abcam). The secondary antibodies used included goat anti-rabbit (1:10,000; sc-2004; Santa Cruz Biotechnology Inc.) and goat anti-mouse (1:10,000; 115-035-003; Jackson ImmunoResearch). Secondary antibody detection was performed by chemiluminescence (SuperSignal® West Pico, Thermo Scientific/Fisher).

### Quantitative real-time reverse transcription PCR

RNA and RT products were prepared as described previously [13,19,23]. Quantitative real-time reverse transcription PCR (qRT-PCR) reactions were performed using TaqMan® gene expression assays (Life Technologies) for mouse *Nr3c1 (GR)* (Mm00433832_m1) *Brca1* (Mm01249840_m1), *Oas2* (Mm00460961_m1), *Ces1* (Mm00491334_m1), *Hsd11b1* (Mm00476182), *Ch25h* (Mm00515486_s1), *Slc5a9* (Mm00523837_m1). Mouse *Tbp* was used as an internal control for all qRT-PCR experiments (Mm00446971_m1; Life Technologies). Quantitative RT-PCR reactions were performed using the SuperScript® III Platinum® One-Step Quantitative RT-PCR system (Invitrogen) with 50–250 ng RNA in triplicate and 1 μL TaqMan® gene expression assay per reaction. The PCR protocol consisted of one cycle of (900 sec at 50°C and 120 sec at 95°C), followed by 40 cycles of (15 sec at 95°C and 30 sec at 60°C), and was run on an Eppendorf Mastercycler®. Gene expression was calculated relative to the results for the untreated or empty vector sample with the comparative *C*_t_ (ΔΔ*C*_t_) method presented by PE Applied Biosystems (Perkin Elmer).

### ChIP assay

EPH-4 cells were plated and treated as described above. ChIP assays were performed with the ChIP-IT™ Express Enzymatic kit (Active Motif, Carlsbad, CA, USA). Each reaction was performed using chromatin from 2 × 10^6^ cells and 2 μg per reaction of affinity-purified antibody (or water as a no antibody negative control). The following antibodies were used: anti-GR (ab3579; Abcam), anti-GABPα (sc-22810; Santa-Cruz), anti-GABPβ (sc-28684; Santa Cruz) anti-haemaglutinin (sc-805; Santa-Cruz), and anti-acetylated histone H3 (06–599; Upstate Biotechnology, Lake Placid, NY, USA). Walking PCR primers were designed to cover approximately 3000 bp of each the *Ch25h*, *Hsd11b1* distal P1, and *Hsd11b1* proximal P2 promoter regions (primers listed in Additional file 1: Tables S2-S4). The PCR protocol consisted of one cycle of 180 sec at 95°C followed by 38 cycles of (30 sec at 95°C, 30 sec at 60°C, 30 sec at 72°C) and a final cycle of 240 sec at 72°C. ChIP DNA was quantified by quantitative PCR using the QuantiTect SYBR Green PCR kit using 2 μL of ChIP DNA and ChIP PCR primers for mouse *Ch25h* “region 11” from position -447 to -118 ((+) 5’-CAACGGACCCAGTACCAGCA and (-) 5’-ACGTAAAGAACTGTTTGCTTGCC. The PCR protocol consisted of one cycle of 900 sec at 94°C followed by 40 cycles of (30 sec at 94°C, 30 sec at 60°C, 30 sec at 72°C).

### Expression microarray

RNA was prepared as described previously [13,19,23] from EPH-4 EV-50 and shGR-19 stable cell lines. The quality of total RNA was determined with an Agilent 2100 Bioanalyzer (Agilent Technologies). The samples were selected for microarray analysis or for qRT-PCR provided that they had an RNA integrity number (RIN) >7.0, a clear gel image, and no DNA contamination observed on the histogram. A total of 300 ng quality-checked total RNA from each sample (in duplicate) was amplified and labeled with Cy3 using the Agilent QuickAmp kit (Agilent Technologies). Cy3 labeling efficiency and amplification efficiency were assessed using a NanoDrop ND-1000 spectrophotometer (NanoDrop Technologies). 1.65 μg of Cy3-labeled cRNA for each sample was hybridized to an Agilent Whole Mouse Genome 4 × 44 K gene expression array (G2519F-014868, Agilent Technologies). After 17 hours of hybridization, arrays were washed and scanned according to the Agilent gene expression array protocol. The data was normalized by the Feature Extraction software (10.5.1.1) with default parameter settings for one-colour oligonucleotide microarrays and then transferred to GeneSpring GX version 9.0.2 (Agilent Technologies) for further statistical evaluation. In GeneSpring, normalization and data transformation steps for one-colour data were applied as recommended by Agilent Technologies. The data were analyzed using GeneSpring, and genes with >2.0 fold differential expression (both increased and decreased; p < 0.01) between EV-50 and shGR-19 were ranked by fold.

Functional analysis of differentially expressed genes from microarray data was performed using the Gene Ontology Enrichment Analysis Software Toolkit (GOEAST) program, which adjusts the raw p-values into a false discovery rate using the Benjamini-Yekutieli method [24]. In addition to classifying genes based on biological process, molecular function, and cellular component ontologies, we employed Ingenuity Pathway Analysis (IPA; http://www.ingenuity.com) to identify biological networks regulated by GR. The upregulated and downregulated gene sets between EPH-4 EV-50 and shGR-19, as well as both differentially expressed sets together, were used for network analysis. Following GeneSpring analysis, Agilent probe set IDs were uploaded into IPA and queried with all other genes stored in the Ingenuity Knowledge Base. In reporting our results, we focused on networks with high IPA network scores, which demonstrate strong evidence for a given biological pathway being regulated by GR. The results of our GeneSpring differential analysis, as well as the GOEAST and IPA functional analyses, were coalesced in order to construct a list of candidate genes that may be regulated similarly to *Brca1*. Five candidate genes exhibiting decreased differential expression between EV-50 and shGR-19 were chosen for validation and subsequent analyses.

### Statistical analysis

The level of GR knockdown in the EPH-4 stable cell lines shGR-73 and shGR-19 (relative to EV-50) was quantified by densitometric analysis of the GR and TBP Western blots using ImageJ. Standard deviation between triplicates from qRT-PCR experiments were calculated according to the ΔΔ*C*_t_ method presented by Applied Biosystems. Standard deviation between triplicates in luciferase assays was calculated using Microsoft Excel 2010. Statistical significance calculations for qRT-PCR experiments and luciferase assays were performed with GraphPad Prism 5 Software, using the unpaired, two-tailed t-test function assuming equal variances of the averaged data.

## Results

### GR and Brca1 levels are decreased in cells stably expressing shGR

**Figure 1:**
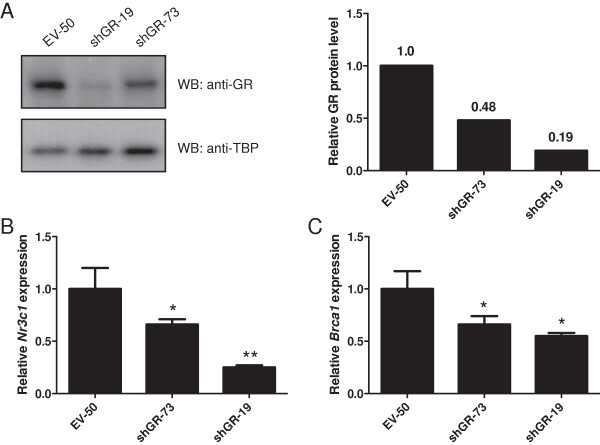
**Expression of GR and Brca1 is decreased in cells stably expressing an shRNA vector against endogenous GR.** EPH-4 cells were stably transfected with a puromycin selectable marker and either an empty vector (H1-2; EV) or an shRNA vector directed against the endogenous glucocorticoid receptor (shGR). Cells were puromycin-selected and expanded. **A**. EV-50, shGR-19, and shGR-73 stable clone lines were lysed and subjected to Western blotting to determine GR expression (shown in left panel). Densitometric analysis was performed to quantify the level of GR protein knockdown in shGR-73 and shGR-19 relative to EV-50 (shown in right panel; numbers indicate protein levels relative to EV-50). **B-C**. RNA was prepared from EPH-4 stable cell lines EV-50, shGR-73, and shGR-19, and qRT-PCR analysis of mouse **B**. *Nr3c1* (GR) and **C**. *Brca1* expression was conducted using TaqMan gene expression assays for each gene. Raw *C*_t_ values for each gene were normalized to raw *C*_t_ values for mouse *Tbp* internal control for triplicate samples, and are presented as the level of expression relative to the EV-50 sample. Bars represent the mean of technical replicates, and error bars represent standard deviation (N = 3). Statistically significant changes in gene expression relative to EV-50 are indicated for each gene: one asterisk, p < 0.05 (significant); two asterisks, p < 0.005 (very significant).

**Figure 2:**
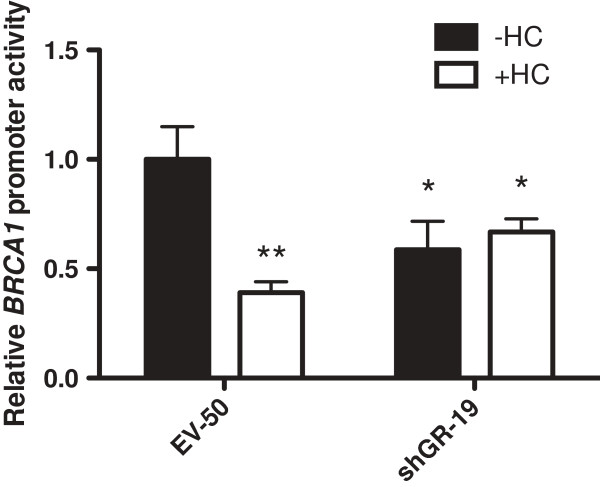
***BRCA1*promoter activity is reduced and no longer repressed in the presence of HC in cells stably expressing shGR.** EPH-4 EV-50 and shGR-19 stable cells were transiently transfected with the L6 *BRCA1* promoter reporter construct, treated 24 hours after transfection with either ethanol vehicle (-HC) or 1 μg/mL HC (+HC), and assayed for luciferase activity following a 48 hour incubation. Bars represent the mean of technical replicates, and error bars represent standard deviation (N = 3). Statistically significant changes in *BRCA1* promoter activity relative to EV-50 (-HC) are indicated: one asterisk, p < 0.05 (significant); two asterisks, p < 0.005 (very significant).

We have previously shown that unliganded GR positively regulates *BRCA1* promoter activity in EPH-4 mouse mammary cells [13]. This effect may be representative of a novel role for unliganded GR as a transcriptional activator of multiple genes in the breast. In order to address this hypothesis as well as study the involvement of unliganded GR in cellular processes, we stably transfected the non-malignant mouse mammary cell line EPH-4 with a short hairpin RNA (shRNA) vector directed against human/mouse GR (shGR) as well as empty H1-2 vector as a control (EV). Protein lysates and RNA were prepared from puromycin-selected clonal isolates maintained in the absence of glucocorticoids, and GR expression was examined by Western blot and qRT-PCR. The stable cell lines shGR-73 and shGR-19 exhibited reduced levels of GR protein (Figure 1A) and expression of *Nr3c1* (GR) mRNA (Figure 1B) relative to the empty vector control cell line EV-50, with shGR-19 exhibiting the greatest degree of GR knockdown at both the protein and mRNA levels. Both shGR-73 and shGR-19 cells displayed reduced endogenous *Brca1* expression compared with EV-50 (Figure 1C), which reflects the positive regulatory effect that GR normally has on this gene. Furthermore, transiently transfecting the *BRCA1* proximal promoter construct L6 resulted in a reduction in its activity by approximately 50% in shGR-19 cells compared to EV-50 cells in the absence of HC, indicating that the level of endogenous GR in these cells is insufficient to positively regulate *BRCA1* expression (Figure 2). In support of this, treatment of shGR-19 cells with HC did not result in any additional repression of *BRCA1* activity.

### Expression microarray analysis

The creation of the stable cell lines EV-50 and shGR-19 afforded us the ability to identify targets exclusively regulated by unliganded GR by comparing gene expression in cells depleted of GR (shGR-19) to that in cells expressing normal endogenous levels of this transcription factor (EV-50). Whole genome expression microarray analysis resulted in the identification of a total of 603 entities (genes or transcripts) with at least a 2-fold change and p < 0.01 between EPH-4 EV-50 and shGR-19 cells, including 260 upregulated genes and 343 downregulated genes in shGR-19 relative to EV-50 (see Additional file 2). The data discussed in this publication have been deposited in NCBI’s Gene Expression Omnibus [25] and are accessible through GEO Series accession number GSE51408 (http://www.ncbi.nlm.nih.gov/geo/query/acc.cgi?acc=GSE51408). Genes upregulated in shGR-19 compared to EV-50 are likely negatively regulated by unliganded GR, since they are increased in the absence of endogenous unliganded GR. In contrast, genes downregulated in shGR-19 compared to EV-50 are positively regulated by unliganded GR, since they are decreased in the absence of endogenous unliganded GR. Among the genes downregulated in shGR-19, the GR gene, *Nr3c1*, was decreased approximately 4-fold, confirming the stability of GR knockdown in this cell line. While *Brca1* did not qualify for the analysis following the 2-fold cutoff, its expression was decreased approximately 1.5-fold in shGR-19, confirming our previous report that GR positively regulates *Brca1* activity, since GR depletion results in decreased expression of endogenous *Brca1*.

#### *Functional analyses*

In order to analyze potential functional trends in our microarray data, we performed functional analyses of the lists of differentially expressed up and downregulated genes. Our Gene Ontology (GO) analysis was completed using GOEAST (Gene Ontology Enrichment Analysis Software Toolkit) [24]. This program enabled the determination of the most highly represented GO categories in response to GR depletion, and the number of genes in each set (up and downregulated) belonging to those categories. This analysis determined that the gene targets negatively regulated by unliganded GR were involved in various developmental processes, while the targets of positive regulation by unliganded GR were involved in processes related to immune system regulation and signaling (see Additional file 3: Figures S1 and S2). Furthermore, there was little to no overlap in GO terms between the two gene lists; while several genes positively regulated by unliganded GR were involved in pro-apoptotic pathways, a number of genes negatively regulated by unliganded GR appeared to be anti-apoptotic. In order to examine the structure of regulatory networks underlying the response to depleted endogenous GR expression, we performed Ingenuity Pathway Analysis using both sets of differentially expressed genes between EPH-4 EV-50 and shGR-19, as well as both differentially regulated gene sets together. Unlike GO analysis, which classifies individual gene candidates based on function, IPA networks represent gene relationships and interactions that are linked to specific molecular and cellular mechanisms.

**Figure 3:**
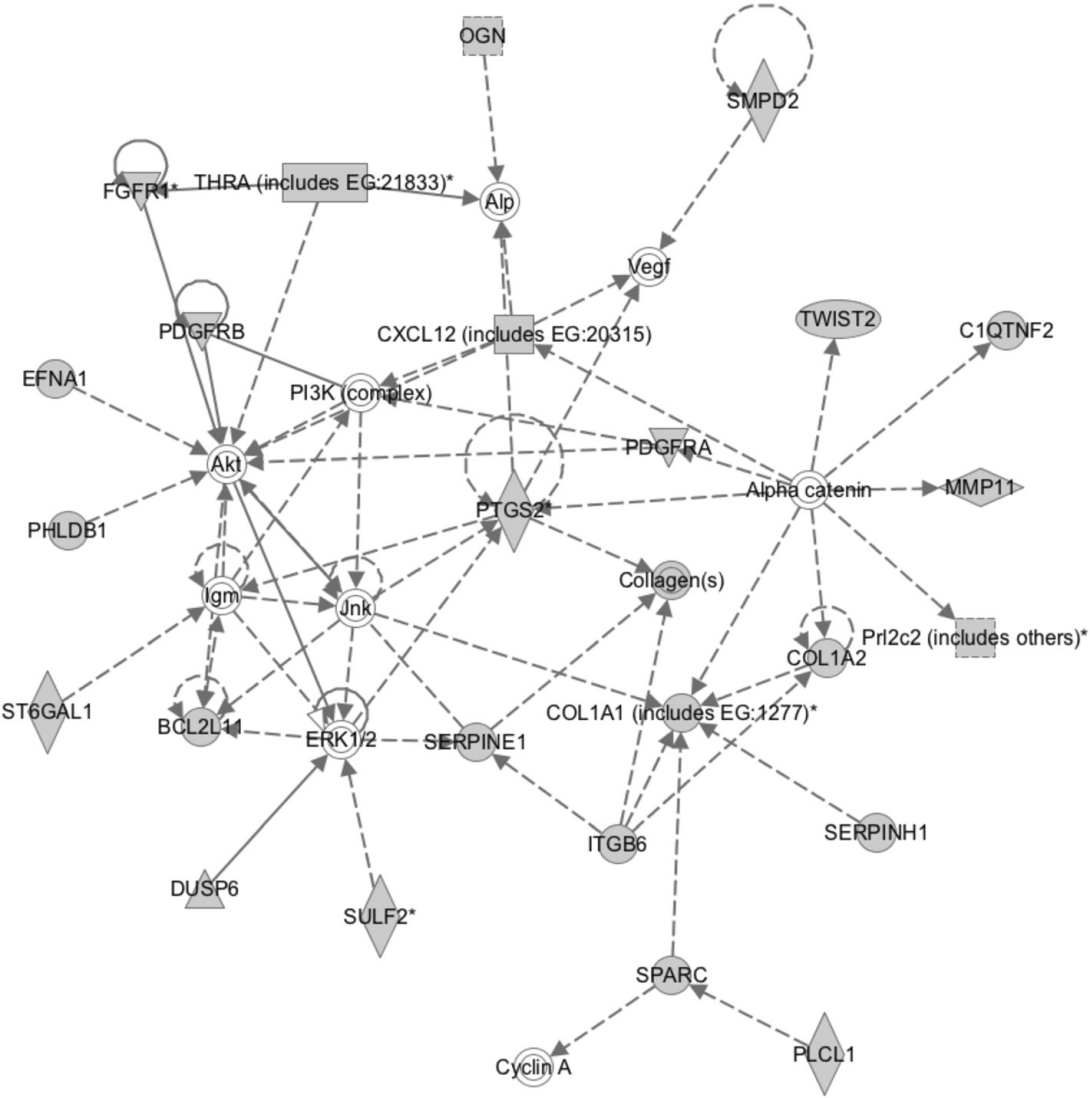
**Ingenuity Pathway Analysis of genes negatively regulated by unliganded GR.** IPA identified this hub as the top signaling network among candidate genes upregulated in EPH-4 shGR-19 compared to EV-50 (IPA network score = 41) *i.e.* negatively regulated by unliganded GR. This network was associated with cardiovascular system development and function, cellular movement, and organismal development. Solid lines indicate a physical interaction between molecules. A solid line with an arrow indicates that one factor acts on the other, while a solid line with a perpendicular bar at the end denotes that one factor inhibits the other. Dotted lines indicate an indirect cellular interaction between factors. Shaded molecules represent candidate genes revealed to be upregulated between EPH-4 EV-50 and shGR-19 in our microarray analysis, while white molecules represent factors imputed from the Ingenuity Knowledge Base. The different shapes of the molecules represent their different functional activities: square, cytokine; circle, other; triangle, kinase/phosphatase; rectangle, G protein coupled receptor; oval, transcription regulator; diamond, enzyme. A full explanation of IPA annotation can be found at http://www.springerimages.com/Images/LifeSciences/1-10.1007_s12014-010-9053-0-2.

IPA revealed that there was a high probability for finding genes that were negatively regulated by unliganded GR in a network hub centered on prostaglandin-endoperoxide synthase 2 (PTGS2; cyclooxygenase (COX)-2) and chemokine signaling (Figure 3). Genes in this “top” network had p-values of <0.05 based on Fisher’s exact test, and were associated with cardiovascular system development and function, cellular movement, and organismal development, indicating that unliganded GR, either directly or indirectly, negatively regulates these processes. This network was composed primarily of gene candidates of negative regulation by unliganded GR identified by our microarray study, as indicated by the shaded entities, while the white entities represent factors imputed from the IPA Knowledge Base (IPA network score = 41).

**Figure 4:**
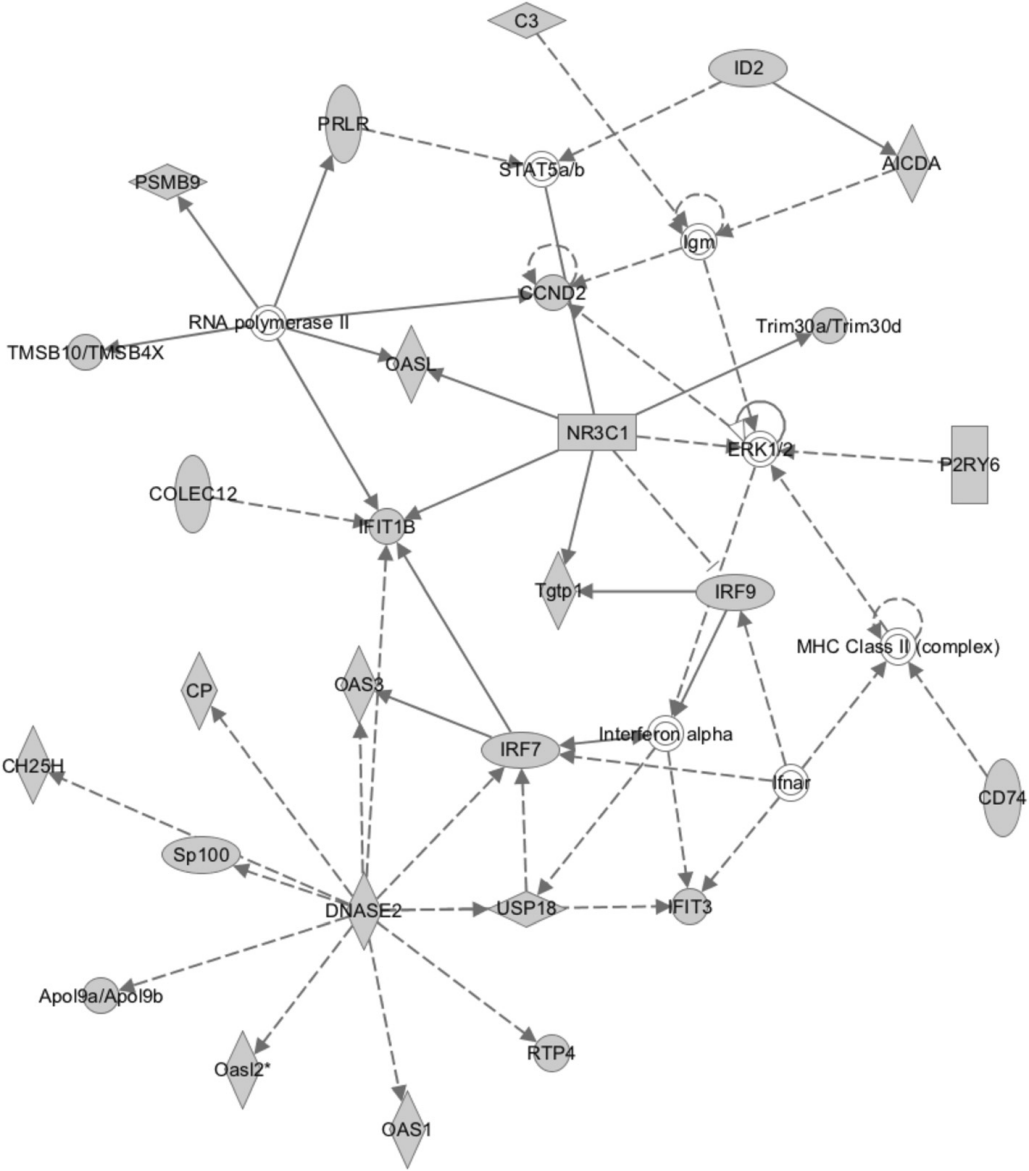
**Ingenuity Pathway Analysis of genes positively regulated by unliganded GR.** IPA identified this hub as a top signaling network among candidate genes downregulated in EPH-4 shGR-19 compared to EV-50 (IPA network score = 41) *i.e*. positively regulated by GR. This network was associated with dermatological diseases and conditions, inflammatory disease, and neurological disease. Annotation of IPA network shapes and molecular relationships as specified in Figure 3.

According to IPA, the genes positively regulated by unliganded GR from our microarray study had a high probability of being found in a “top” network signaling hub involving interferon and immune system signaling. Genes in this network, with p-values of <0.05, were associated with dermatological diseases and conditions, inflammatory disease, and neurological disease (Figure 4). This network was comprised almost entirely of gene candidates of positive regulation by unliganded GR identified by the present study (IPA significance score = 41). The GR gene (*Nr3c1*), which was downregulated approximately 4-fold in shGR-19, was central in this pathway, establishing its direct involvement in the positive regulation of these processes. Also prominently featured in this network were Cyclin D2 (*CCND2*), and members of the oligoadenylate synthetase (*Oas*), interferon regulatory factor (*Irf*), and interferon-induced tetratricopeptide repeat (*Ifit*) families of genes. When all entities (*ie*. both lists of candidates of negative and positive regulation by unliganded GR) were analyzed together, there was a high probability for finding candidate genes from the present study in a network hub involving interferon and immune signaling (data not shown). Notably, this pathway consisted primarily of genes from the top network involving candidates that were downregulated in shGR-19 relative to EV-50, including *Nr3c1* itself, suggesting that these biological signaling pathways predominantly involve factors that are positively regulated by unliganded GR. We hypothesized that these factors may be positively regulated by unliganded GR through the same mechanism as we have previously identified for *Brca1*. As such, we focused primarily on this set of genes, and selected five candidates for validation and further analysis.

#### *Candidate gene selection*

Five candidate genes were selected for microarray validation and further characterization based on the combined results of the GeneSpring differential analysis and both the GOEAST (see Additional file 3) and IPA functional analyses, and included *Hsd11b1*, *Ch25h*, *Ces1*, *Oas2*, and *Slc5a9*. Each of these genes was among the top 50 candidates that exhibited at least 10-fold downregulated expression in shGR-19 compared to EV-50. The *Hsd11b1* gene encodes the enzyme 11β-hydroxysteroid dehydrogenase type 1, which is responsible for the interconversion of glucocorticoids between inactive cortisone and active cortisol in humans and between inactive 11-dehydrocorticosterone and active corticosterone in rodents [26]. *Ch25h* encodes the enzyme cholesterol 25-hydroxylase, which catalyzes the synthesis of 25-hydroxycholesterol from cholesterol and molecular oxygen [27], and has a role in the regulation of the innate immune system, where its expression is induced in the presence of TLR ligands [28,29]. The enzyme carboxylesterase 1 is encoded by the *Ces1* gene, which is a serine esterase that hydrolyzes aromatic and aliphatic esters and thus maintains the level of free lipids within cells by monitoring cholesterol esterification levels [30]. The *Oas2* gene encodes 2′,5’-oligoadenylate synthetase 2, which is a member of a family of essential proteins involved in the innate immune response to viral infection [31]. Oas2 is induced by interferons to synthesize 2′,5’-oligoadenylates, which activate latent RNase L, resulting in viral RNA degradation and the inhibition of viral replication [32]. *Oas2* was one member of several *Oas* genes that appeared to be positively regulated by unliganded GR (*Oas1a*, *Oas1c*, *Oas3*, *Oasl1*, *Oasl2*). The protein encoded by *Slc5a9* is a sodium-dependent glucose transporter that is essential for the transport of mannose, 1,5-anhydro-D-glucitol, and fructose [33]. *Slc5a9* was representative of a larger group of solute carrier genes that appeared in our gene set comprised of targets of unliganded GR positive regulation (*Slc23a3*, *Slc39a4*, *Slc46a1*, *Slc7a4*).

#### *Candidate gene validation*

**Figure 5:**
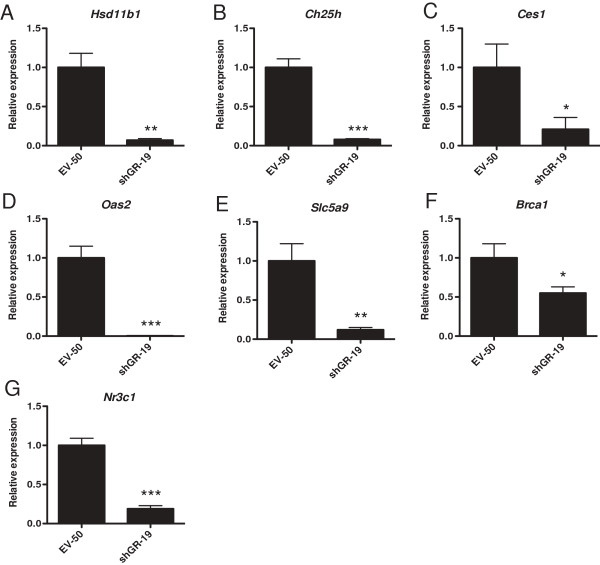
**Validation of microarray candidate genes.** qRT-PCR validation of microarray candidate gene expression was conducted using RNA prepared from EPH-4 EV-50 and shGR-19 stable cells and TaqMan mouse gene expression assays for each gene: **A**. *Hsd11b1*, **B**. *Ch25h*, **C**. *Ces1*, **D**. *Oas2*, **E**. *Slc5a9*, **F**. *Brca1*, and **G**. *Nr3c1*. Raw *C*_t_ values for each gene were normalized to raw *C*_t_ values for mouse *Tbp* internal control for triplicate samples, and are presented as the level of expression relative to the EV-50 sample. Bars represent the mean of technical replicates, and error bars represent standard deviation (N = 3). Statistically significant changes in gene expression relative to EV-50 are indicated for each gene: one asterisk, p < 0.05 (significant); two asterisks, p < 0.005 (very significant); three asterisks, p < 0.0005 (very highly significant).

To validate the microarray, quantitative real-time reverse transcription PCR (qRT-PCR) was performed to assess the mRNA expression of the five selected genes that exhibited decreased differential expression between EPH-4 EV-50 and shGR-19 cells, *Hsd11b1*, *Ch25h*, *Ces1*, *Oas2*, and *Slc5a9*, as well as *Brca1* and *Nr3c1* (Figure 5A-G). Microarray and qRT-PCR resulted in similar levels (*ie*. relative expression in shGR-19 compared to EV-50) of downregulation for all of these genes, confirming the reliability of our microarray results at the mRNA level (Table 1). It is of note that while *Hsd11b1*, *Ch25h*, and *Slc5a9* were expressed at relatively high levels (*ie*. raw *C*_t_ values were lower than those of the housekeeping gene, *Tbp*), both *Ces1* and *Oas2* were expressed less abundantly (*ie*. raw *C*_t_ values were higher than those of *Tbp*, and in the case of *Ces1*, values approached the maximum number of PCR cycles).

**Table 1:** Relative expression of candidate genes in EPH-4 shGR-19 RNA compared to EV-50 RNA in expression microarray vs. qRT-PCR experiments

	**Relative expression: shGR-19 vs. EV-50**	
**Gene**	**Expression microarray**	**qRT-PCR validation**
*Hsd11b1*	0.11	0.07
*Ch25h*	0.10	0.08
*Ces1*	0.10	0.21
*Oas2*	0.02	0.004
*Slc5a9*	0.07	0.12
*Brca1*	0.77	0.55
*Nr3c1*	0.26	0.19

The fold change output for each gene between shGR-19 and EV-50 from the microarray data was converted to relative expression by taking the base-2 logarithm of the absolute fold change and setting this value as the negative exponent of 2. The relative expression values for each gene between shGR-19 and EV-50 in the qRT-PCR were obtained directly during the experimental analysis (ΔΔ*C*_t_ method).

### Expression of candidate genes in response to hydrocortisone and RU-486 treatment

**Figure 6:**
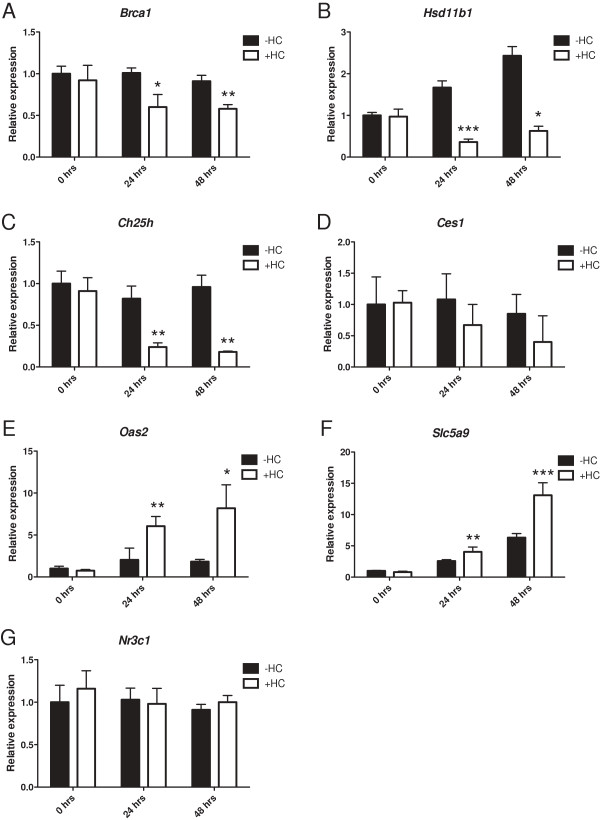
**Expression of microarray candidate genes in response to HC treatment.** EPH-4 cells were treated 24 hours after plating (*ie*. at 0 hrs) with either ethanol vehicle (-HC) or 1 μg/mL HC (+HC) in serum-free media for a period of 48 hours. RNA was prepared at 0, 24, and 48 hours, and qRT-PCR analysis of microarray candidate gene expression was conducted using TaqMan mouse gene expression assays for each gene: **A**. *Brca1***B**. *Hsd11b1*, **C**. *Ch25h*, **D**. *Ces1*, **E**. *Oas2*, **F**. *Slc5a9*, and **G**. *Nr3c1*. Raw *C*_t_ values for each gene were normalized to raw *C*_t_ values for mouse *Tbp* internal control for triplicate samples, and are presented as the level of expression relative to the -HC sample at 0 hrs. Bars represent the mean of technical replicates, and error bars represent standard deviation (N = 3). Statistically significant changes in gene expression relative to the -HC sample for each time point are indicated for each gene: one asterisk, p < 0.05 (significant); two asterisks, p < 0.005 (very significant); three asterisks, p < 0.0005 (very highly significant).

To investigate whether ligand binding to GR exerts the same effect on our candidate genes as we have previously reported for *BRCA1*, we investigated the expression of each gene in the presence of HC. EPH-4 cells were treated with HC, and RNA was prepared at 0, 24, and 48 hours for qRT-PCR analysis. As expected, treatment with HC downregulated *Brca1* by about 50% at both 24 and 48 hours (Figure 6A). Expression of *Hsd11b1* was reduced by about 60% after 24 hours of HC treatment, and by 40% at 48 hours (Figure 6B). Notably, *Hsd11b1* expression increased nearly 3-fold between 0 and 48 hours in untreated (-HC) cells. HC treatment resulted in a decrease in *Ch25h* expression by approximately 80% after 24 and 48 hours (Figure 6C). *Ces1* expression was also downregulated by HC at both 24 and 48 hours, but large standard deviations indicate that this gene may not be expressed at adequate levels (Figure 6D). Expression of *Oas2* increased with HC treatment, by 6- and 8-fold at 24 and 48 hours, respectively (Figure 6E). *Slc5a9* was also positively affected by HC treatment, with expression of this gene increasing 4-fold after 24 hours and 13-fold after 48 hours (Figure 6F). Expression of *Slc5a9* also increased in untreated cells, but this effect was less dramatic than in HC-treated cells. Since HC appears to have an upregulating effect on *Oas2* and *Slc5a9* expression rather than a repressing effect, it is possible that these genes are not regulated by unliganded GR through precisely the same mechanism as *Brca1*. Instead, it is possible that unliganded GR may be poised on a GRE within these promoters but may not activate transcription until ligand binding, making these genes HC-responsive. Expression of *Nr3c1* was unaltered by HC treatment (Figure 6G), indicating GR does not regulate itself in this context.

**Figure 7:**
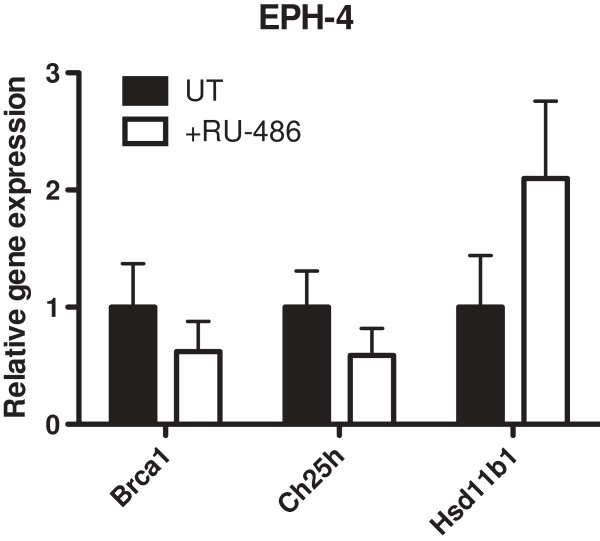
**Expression of microarray candidate genes in response to RU-486 treatment.** EPH-4 cells were treated 24 hours after plating (*ie*. at 0 hrs) with either ethanol vehicle (UT) or 10 μM RU-486 (+RU-486) in serum-free media for a period of 48 hours, after which RNA was prepared. qRT-PCR analysis of gene expression was conducted using TaqMan mouse gene expression assays for *Brca1*, *Hsd11b1*, and *Ch25h*. Raw *C*_t_ values for each gene were normalized to raw *C*_t_ values for mouse *Tbp* internal control for triplicate samples, and are presented as the level of expression relative to the UT sample. Bars represent the mean of technical replicates, and error bars represent standard deviation (N = 3).

We further evaluated the effect of ligand binding on the expression of both *Hsd11b1* and *Ch25h* with the use of the GR antagonist, mifepristone (RU-486). RU-486 is a hydrocortisone analogue that is able to bind to GR but inhibits the transcription of GR target genes [34]. We have previously demonstrated that treatment of mouse mammary cells with RU-486 results in decreased expression of endogenous *Brca1*, indicating that ligand binding, even of a nonsignaling ligand, to GR is the key physiological stimulus for decreased *Brca1* expression, and we suggested that in addition to blocking normal signaling of liganded GR, the repressive ability of RU-486 may be partly due to its ability to disrupt unliganded GR binding at the promoters of its target genes [13]. In the present study, treatment of EPH-4 cells with RU-486 resulted in decreased expression of both *Brca1* and *Ch25h*, but not *Hsd11b1*, which was activated (Figure 7), suggesting that the regulation of these genes may involve different mechanisms of GR signaling.

### The unliganded glucocorticoid receptor interacts directly with the Ch25h promoter and upregulates Ch25h activity

**Figure 8:**
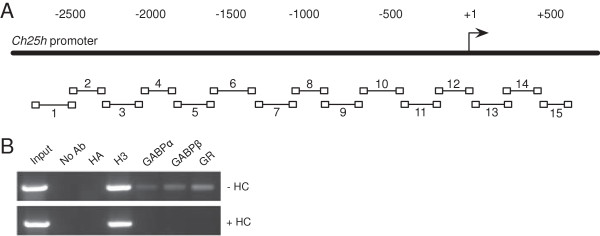
**GR physically interacts with the*Ch25h*promoter in the absence of ligand. A**. Schematic of walking ChIP primers covering ~2800 bp upstream and ~500 bp downstream of the transcription start site of *Ch25h*. The thick black line indicates the *Ch25h* promoter. The arrow and +1 indicate the location of the transcription start site. The numbers above indicate the bp location on *Ch25h*. The boxes below indicate the locations of the ChIP primers, and the region amplified by each primer set is indicated by a line between the boxes. The numbers above and below these lines indicate the designated “region” of the *Ch25h* promoter covered by each primer set. **B**. Chromatin was prepared from EPH-4 non-malignant mouse mammary cells 24 hours after treatment with either ethanol vehicle (-HC) or 1 μg/mL HC (+HC) in serum-free medium. ChIP analysis was carried out with the indicated antibodies and PCR primers amplifying the region between -447 to -219 bp of the *Ch25h* promoter. Ab, antibody; HA, hemagglutinin; H3, acetylated histone H3.

Our microarray and further validation experiments indicated that *Hsd11b1* and *Ch25h* were positively regulated by unliganded GR in the absence of HC and that their expression decreased upon HC addition. In order to determine whether unliganded GR physically interacts with the promoters of these genes, we performed ChIP assays using untreated and HC-treated EPH-4 chromatin and assayed both the *Hsd11b1* and *Ch25h* promoter regions using walking sets of ChIP primers that covered approximately 2800 bp upstream and 500 bp downstream of the defined transcription start sites in both genes (*Ch25h* promoter depicted in Figure 8A). These experiments revealed that GR bound specifically to “region 11” between -447 and -219 bp of *Ch25h* in the absence of HC, and that this interaction was abolished in the presence of HC (Figure 8B). Furthermore, ChIP of the *ets* family members GABPα and GABPβ demonstrated that both of these proteins interacted with this same region of *Ch25h* in the absence but not the presence of HC. This result is of interest since the mechanism by which unliganded GR upregulates *BRCA1* expression involves a protein-protein interaction with GABPβ at the *BRCA1* promoter. The ChIP DNA samples were analyzed via quantitative PCR using the ChIP primers for *Ch25h* “region 11” and values obtained reflect the pattern of band intensities on the ChIP gel (Table 2). Neither GR nor GABP bound to either the *Hsd11b1* P1 (distal) or P2 (proximal) promoters in the absence or presence of HC (data not shown).

**Table 2:** Analysis of ChIP DNA products by quantitative PCR

		**EPH-4 chromatin**	
		**-HC**	**+HC**
	**Input**	26.58	27.06
	**No Ab**	>40	>40
ChIP	**HA**	>40	>40
DNA	**H3**	27.94	28.13
Sample	**GABPα**	32.67	>40
	**GABPβ**	33.5	>40
	**GR**	32.5	>40

The ChIP DNA products were analyzed via quantitative PCR using the ChIP primers for *Ch25h* “region 11” and expressed as relative *C*_t_ values.

**Figure 9:**
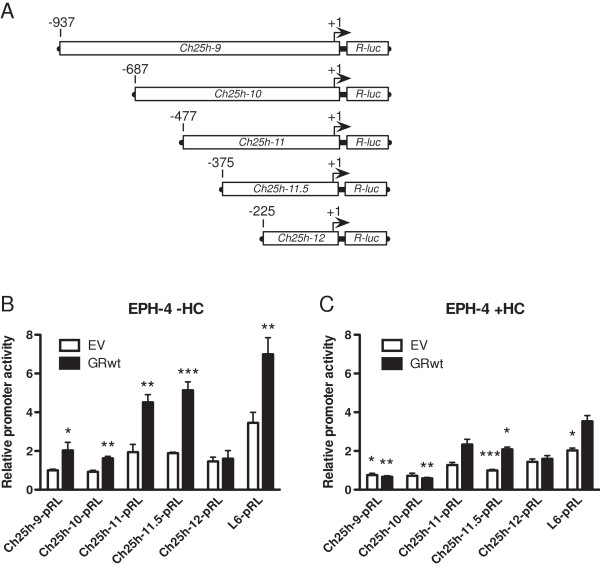
**GR activates the*Ch25h*promoter region between -375 and -225 bp only in the absence of HC. A**. Schematic of *Ch25h* promoter fragments cloned into the pRL-Null vector upstream of the *Renilla* luciferase (*R-luc*) gene. **B**-**C**. EPH-4 cells were transiently transfected with the *Ch25h* promoter reporters Ch25h-9-pRL, Ch25h-10-pRL, Ch25h-11-pRL Ch25h-11.5-pRL Ch25h-12-pRL, and the L6-pRL *BRCA1* promoter reporter, as well as empty vector (EV) or wild-type GR (GRwt) expression vector. Cells were treated 24 hours after transfection with either **B**. ethanol vehicle (-HC) or **C**. 1 μg/mL HC (+HC) in serum-free medium and assayed for luciferase activity following a 48 hour incubation. Bars represent the mean of technical replicates, and error bars represent standard deviation (N = 3). For **B**., statistically significant changes in *Ch25h* promoter activity relative to the EV control for each reporter are indicated: one asterisk, p < 0.05 (significant); two asterisks, p < 0.005 (very significant); three asterisks, p < 0.0005 (very highly significant). For **C**., statistically significant changes in *Ch25h* promoter activity in response to EV and GRwt transfections are indicated relative to the EV transfection for each reporter from the corresponding -HC experiment in **B**.

**Figure 10:**
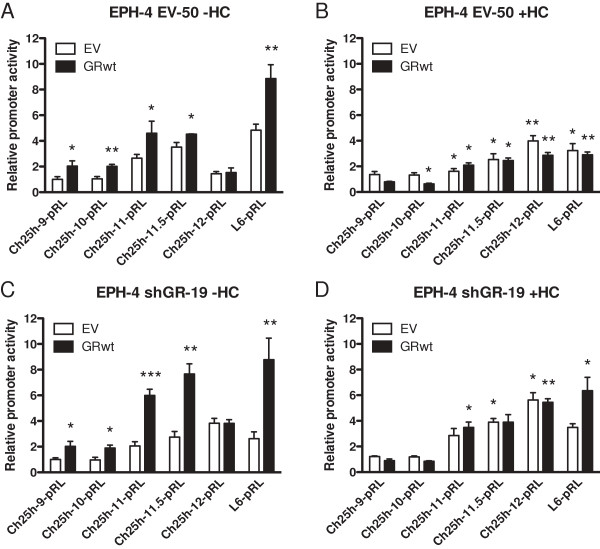
**GR activates the*Ch25h*promoter region between -375 and -225 bp only in the absence of HC in EPH-4 clone cell lines EV-50 and shGR-19.** EPH-4 clone cell lines **A**-**B**. EV-50 and **C**-**D**. shGR-19 were transiently transfected with the *Ch25h* promoter reporters Ch25h-9-pRL, Ch25h-10-pRL, Ch25h-11-pRL Ch25h-11.5-pRL Ch25h-12-pRL, and the L6-pRL *BRCA1* promoter reporter, as well as empty vector (EV) or wild-type GR (GRwt) expression vector. Cells were treated 24 hours after transfection with either **A** and **C**. ethanol vehicle (-HC) or **B** and **D**. 1 μg/mL HC (+HC) in serum-free medium and assayed for luciferase activity following a 48 hour incubation. Bars represent the mean of technical replicates, and error bars represent standard deviation (N = 3). For **A** and **C**., data was normalized to the EV control in the Ch25h-9-pRL transfection (*ie*. separately for each cell line). Statistically significant changes in *Ch25h* promoter activity relative to the EV control for each reporter are indicated: one asterisk, p < 0.05 (significant); two asterisks, p < 0.005 (very significant); three asterisks, p < 0.0005 (very highly significant). For **B** and **D**., data was normalized to the EV control in the Ch25h-9-pRL transfection from the corresponding -HC experiments in **A** and **C**. Statistically significant changes in *Ch25h* promoter activity in response to EV and GRwt transfections are indicated relative to the EV transfection for each reporter from the corresponding -HC experiments in **A** and **C**.

We previously reported that transfection of a GR expression vector increased *BRCA1* activity in transient transfection assays with the proximal *BRCA1* promoter reporter. In order to investigate the effect of GR on *Ch25h* promoter expression, we transfected EPH-4 cells with *Ch25h* reporter constructs of varying lengths: Ch25h-9 (-937), Ch25h-10 (-687), Ch25h-11 (-477), and Ch25h-11.5 (-375), which each contained Ch25h “region 11”, either in its entirety or the proximal half, and Ch25h-12 (-225), which did not contain this region (Figure 9A). This transfection was performed in the absence (Figure 9B) and presence of HC (Figure 9C). In the absence of HC, luciferase activity significantly increased for all reporters containing this site following the addition of exogenous GR (GRwt), while it did not increase for Ch25h-12 (Figure 9B). The level of increase in activity of Ch25h-9, Ch25h-10, Ch25h-11, and Ch25h-11.5 in response to exogenous GR addition was comparable to that of *BRCA1* (L6) (approximately 50% increase in relative promoter activity in response to GR addition). Treatment with HC resulted in downregulation of *BRCA1* L6 and all *Ch25h* promoter reporters containing the region between -375 and -225 bp compared to the activity of each observed in the -HC experiment, during transfection with both EV and GRwt (Figure 9C). Conversely, HC addition had no effect on the activity of Ch25h-12 compared to the -HC experiment, indicating that this region is not HC-responsive. The effect of exogenous GR addition on the activity of *Ch25h* was also investigated using the EPH-4 clone cell lines EV-50 and shGR-19 in both the absence and presence of HC. Consistent with the wild-type EPH-4 transfection, activity of the L6 *BRCA1* promoter and all *Ch25h* reporters containing the region between -375 and -225 bp significantly increased following the addition of exogenous GR (GRwt) in the absence of HC in both EV-50 and shGR-19 cell lines, while activity of Ch25h-12 was unaltered by GR addition (Figures 10A and C). The presence of HC had a repressive effect during EV and GRwt transfections with L6 *BRCA1* and some of the *Ch25h* reporters in EV-50, but not shGR-19 cells, emphasizing the difference in endogenous GR levels between these cell lines (Figures 10B and D). Unlike in the wild-type EPH-4 transfection, the activity of Ch25h-12 was slightly upregulated in response to HC during transfection with both EV and GRwt in both EPH-4 clone cell lines (Figures 10B and D). While normalization in this experiment was performed separately within cell lines, it is of note that the *Ch25h* reporters were approximately 50% less active in shGR-19 cells than in EV-50 cells in the -HC experiment (*ie*. if the cell lines were compared directly to one another). Collectively, these assays support the existence of a response element for unliganded GR within the *Ch25h* promoter “region 11” and that the minimal region required is between -375 and -225 bp.

**Figure 11:**
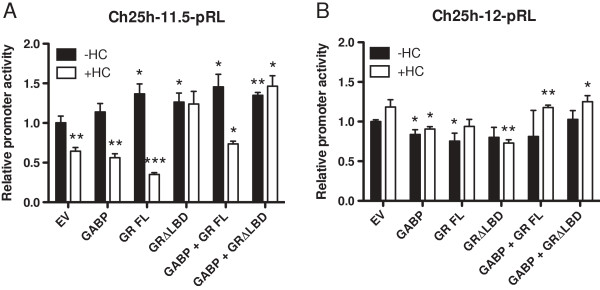
**GRΔLBD activates the*Ch25h*promoter in the presence and absence of HC.** EPH-4 cells were transiently transfected with the *Ch25h* promoter reporters **A**. Ch25h-11.5-pRL and **B**. Ch25h-12-pRL as well as with expression vectors for GABPα/β (GABP), full-length GR (GR FL) and GR lacking the ligand binding domain (GRΔLBD). Cells were treated 24 hours after transfection with either ethanol vehicle (-HC) or 1 μg/mL HC (+HC) and assayed for luciferase activity following a 48 hour incubation. Bars represent the mean of technical replicates, and error bars represent standard deviation (N = 3). Statistically significant changes in *Ch25h* promoter activity relative to the EV (-HC) transfection are indicated: one asterisk, p < 0.05 (significant); two asterisks, p < 0.005 (very significant).

In order to demonstrate that unliganded GR is responsible for the observed activation of *Ch25h*, we transiently transfected EPH-4 cells with the *Ch25h* promoter reporters Ch25h-11.5-pRL (which contains the predicted minimal GR binding region between -375 and -225 bp) and Ch25h-12-pRL (does not contain the GR binding region) along with full length GR (GR FL) and a GR mutant lacking the entire ligand binding domain (GRΔLBD), each in the absence and presence of HC treatment. Both GR FL and GRΔLBD activated Ch25h-11.5-pRL in the absence of ligand (Figure 11A). While activation by GR FL was abolished upon the addition of HC, activation by GRΔLBD was sustained in the presence of HC, emphasizing that in contrast to the wild-type protein, it is now immune to the effects of HC. This mutant is unable to bind ligand, but can still maintain its interaction with the *Ch25h* promoter and activate its expression. Similar results were obtained with the *Ch25h* promoter reporters Ch25h-9-pRL, Ch25h-10-pRL, and Ch25h-11-pRL (see Additional file 4: Figure S3). Neither GR FL nor GRΔLBD was able to activate the construct lacking the GR responsive region (Ch25h-12-pRL), and its activity was not significantly altered upon HC addition (Figure 11B), adding further support for the existence of a response element for unliganded GR contained within the -375 to -225 bp region of *Ch25h*. The presence of GABP alone or in combination with the GR expression vectors did not have a significant effect on the activity of either Ch25h-11.5-pRL or Ch25h-12-pRL. These experiments corroborate our previously described model of gene regulation by unliganded GR in which GR binds to a gene promoter in the absence of HC to activate expression, and in the presence of HC, the activity of the gene decreases due to the dissociation of GR from the promoter.

## Discussion

We have previously shown that unliganded GR is a positive regulator of *BRCA1* expression, and that the presence of ligand negates this regulation. Here, we have continued to explore the role of unliganded GR in the breast, and report the first identification of potential targets of unliganded GR. Expression microarray analysis revealed 343 genes that were positively regulated by unliganded GR, thus illuminating a previously unknown role for unliganded GR in the regulation of a network of genes and adding a new dimension to the GR signaling pathway. We selected five targets of positive regulation by unliganded GR for validation and further analysis. Both *Ch25h* and *Hsd11b1* were repressed by the addition of HC, and *Ch25h* appeared to be regulated by unliganded GR through a similar mechanism as that reported for *Brca1. Oas2* and *Slc5a9* appeared to be activated by both unliganded and liganded GR and may represent a different class of unliganded GR targets.

In the current study, the expression patterns of *Ch25h* indicate that it is regulated similarly to *Brca1* in both the presence and absence of HC. The Ch25h enzyme is responsible for converting cholesterol into 25-hydroxycholesterol, which has been shown to inhibit cell growth and induce apoptosis [35]. The *Ch25h* gene is present in the majority of vertebrate species, being expressed at low levels in brain, lung, heart, and kidney tissues, but is absent from lower organisms such as yeast and flies [36]. While *Ch25h* gene expression is low in resting immune cells, it is induced several hundred-fold when cells are activated with various toll-like receptor (TLR) ligands, suggesting a role for this enzyme in immune system regulation [28,29]. According to IPA analysis, *Ch25h* appeared in the top network signaling hub regulated by unliganded GR that was centered on immune system and inflammatory signaling. In this network, *Ch25h* shared indirect interactions with various factors known to be involved in pro-apoptotic pathways, such as *Dnase2a*[37], as well as several members of the *Irf* and *Oas* families, which were also found by our microarray as targets of unliganded GR.

In support of our previously reported model of unliganded GR as a positive regulator of gene expression, we found that GR physically interacted with a specific region (between -477 to -219 bp) of the *Ch25h* promoter in the absence of ligand, while the addition of HC abolished this interaction. Furthermore, the activity of various *Ch25h* reporters containing the region between -375 and -225 bp increased following the addition of exogenous GR in the absence of ligand, while GR addition had no effect on a *Ch25h* reporter that lacked this region. Analysis of predicted transcription factor binding sites by Alibaba2.1 (http://www.gene-regulation.com) did not reveal any GRE sites within this sequence. Collectively, these results suggest that *Ch25h* is regulated by unliganded GR through a similar molecular mechanism as we have described for *BRCA1*[13].

The *Hsd11b1* gene encodes the enzyme Hsd11b1, which is responsible for controlling the biological activity of glucocorticoids in target tissues. *Hsd11b1* is extensively expressed, particularly in metabolic tissues such as liver, muscle, and adipose [38]. This enzyme is involved in mechanisms of both innate and acquired immune system modulation, with its expression being enhanced in response to a variety of cytokines and inflammatory stimuli [39,40]. Accordingly, IPA analysis revealed that *Hsd11b1* was associated with a network involving several of the same factors as those appearing in the signaling hub with *Ch25h*, including *Dnase2a* and several members of the *Irf* and *Oas* gene families (data not shown). However, this second network was associated with a slightly lower IPA network score, implying more extrapolated connections between our gene set and the identified network. Similar to *Brca1* and *Ch25h*, *Hsd11b1* expression was negatively regulated by HC. However, *Hsd11b1* expression was not repressed by treatment with RU-486, and our ChIP experiments did not show evidence of GR binding to either the distal P1 or proximal P2 promoters of the *Hsd11b1* gene in the absence (or presence) of ligand (data not shown), which may indicate that this gene is either regulated by unliganded GR through an alternate indirect mechanism, or that this interaction occurs outside the region defined by our ChIP primers.

Expression of both *Oas2* and *Slc5a9* was decreased when GR was depleted but in contrast to *Ch25h* and *Hsd11b1*, these genes were significantly activated by HC addition. We suggest that in the absence of hormone, these genes are bound by unliganded GR, where it contributes to the positive regulation of these genes as observed in our microarray analysis. During HC treatment, GR remains bound to the promoter, perhaps via a different protein complex or through a canonical GRE. This offers an explanation for the HC-responsiveness of both *Oas2* and *Slc5a9*, which each display kinetics characteristic of a canonical GRE in response to glucocorticoid binding, such as the *IκB-α* gene, which is induced 23-fold in response to dexamethasone [41]. Promoter analysis using Alibaba2.1 revealed that both *Oas2* and *Slc5a9* contain one or more GRE consensus sequences within their promoter regions. While the binding of unliganded GR to a canonical GRE has not been reported thus far, ChIP-seq analysis of GR binding in A549 lung cells has previously revealed approximately 2600 genes that are weakly bound by unliganded GR [17], representing a mechanism through which GR upregulates genes both in the absence and presence of hormone. This theory merits further investigation.

Beyond our candidate gene analysis, GOEAST and IPA functional analyses revealed that a number of genes positively regulated by unliganded GR were involved in pro-apoptotic pathways, including *Dnase2a*, *Casp1*, *Casp4*, *Card11*, *Xaf1*, *Hsh2d*, and multiple members of the *Irf* and *Tnf* family of genes. In contrast, the genes negatively regulated by unliganded GR appeared to be involved in various developmental and morphogenetic processes, and several of these were involved in anti-apoptotic processes, such as *Faim3*, *Bcl7c*, *Bcl2l11*, *Smad6*, *Atf5*, and *Adora1*. Among the targets of positive regulation by unliganded GR included several Interferon Regulatory Factors (*Irfs*) and members of the 2′,5’-oligoadenylate synthetase (*Oas*) gene family, which are collectively induced in response to interferons (IFNs) [42-44]. IFN-inducible genes are often associated with apoptotic pathways, and some of these factors have been reported to be regulated by BRCA1 [45], which is known to participate in the maintenance of genomic integrity through mediation of both DNA repair and apoptosis mechanisms in the breast [46,47]. A number of other BRCA1-related factors known to participate in DNA repair and apoptotic events, such as *Brca2*[48], *Fancd2*[49], and *Recql*[50], were positively regulated by unliganded GR. It is possible that *BRCA1* is central in the network of genes upregulated by unliganded GR. BRCA1 has recently been found to upregulate the activity of phosphorylated GR, and this activation was required for GR autoregulation [51]. It is possible that a cooperative feedback loop exists between BRCA1 and GR, whereby levels of BRCA1 predict levels of GR, and vice versa, and this may represent a mechanism of regulating basal levels of unliganded GR within the breast.

**Figure 12:**
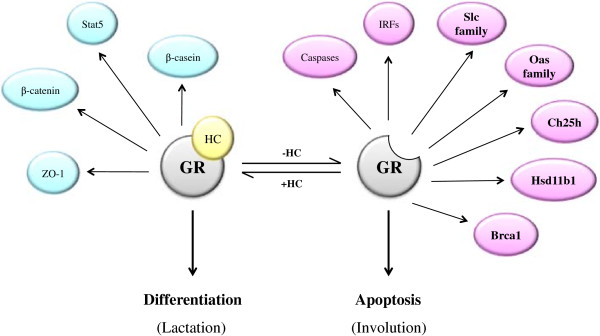
**Summary of signaling networks and pathways regulated by the glucocorticoid receptor.** Targets of liganded and unliganded GR appear to possess opposing functions in the breast. While liganded GR is involved in maintaining functional differentiation, such as occurs during lactation, several unliganded GR targets appear to be pro-apoptotic, and may be involved in involution. Factors in blue represent known targets of liganded GR signaling. Factors in purple represent targets revealed by our microarray to be positively regulated by unliganded GR. Targets in bold purple represent candidates investigated during our microarray validation.

It is well established that glucocorticoids and liganded GR are required for the growth and maintenance of the mammary gland, as well as the suppression of apoptosis during functional differentiation [52]. However, during quiescence and involution, glucocorticoid levels are at a minimum, which suggests a role for unliganded GR in these processes [52,53] (Figure 12). Maintenance of the quiescent adult breast is not dependent upon glucocorticoids, and, as a result, experiences limited proliferation and differentiation [53]. During this period, targets of unliganded GR are upregulated, and the higher level of pro-apoptotic factors produced could be responsible for helping to clear abnormal cells. Levels of intracellular glucocorticoids, particularly cortisol, rise gradually during pregnancy [54-57], where the breast experiences extensive proliferation of the terminal ductal lobuloalveolar units (TDLUs) [58]. At parturition, cortisol levels rise dramatically [54,57], and the breast undergoes functional differentiation of the TDLUs, marking the initiation of lactation [59]. Liganded GR is known to support acinus formation and spatial organization during pregnancy and lactation by regulating the expression of proteins required for maintenance of tight junctional complexes, such as adherens junctions proteins ZO-1 and β-catenin [12,60,61]. Liganded GR also upregulates signal transducer and activator of transcription 5 (STAT5) and enhances β-casein gene transcription during lactation [62-64]. Signaling by liganded GR in this manner maintains a state of terminal differentiation in the breast, both by upregulating the expression of the above-mentioned genes involved in breast morphogenesis, and negating signaling by unliganded GR, thus preventing apoptosis and the onset of involution. Decreased *Brca1* expression reported during lactation in the mouse mammary gland may be reflective of this loss of unliganded GR activation [53]. The cessation of suckling initiates a decrease in circulating cortisol levels, which induces post-lactational regression (involution), an apoptotic process whereby the breast reverts to a quiescent, pre-pregnancy state [3,65]. During involution, signaling by liganded GR is lost, and signaling by unliganded GR would be re-established, thus upregulating pro-apoptotic factors to encourage involution, and repressing factors involved in differentiation. Accordingly, the decrease in cortisol associated with induction of involution coincides with an increase in *Brca1* expression in mice [53].

While ligand-independent activity has been previously reported for other nuclear receptors, including GR, this activity has been in response to other stimuli [14,66-69]. In contrast, our previous and current work indicates that unliganded GR constitutively regulates basal expression of its target genes, which suggests that the endogenous levels of unliganded GR itself may directly determine the expression level of these genes. In the quiescent breast, where maintenance of this state is not dependent upon glucocorticoids, the greater availability of unliganded GR is postulated to increase pro-apoptotic signaling, which could result in the elimination of abnormal cells. Unliganded GR thus offers protection from tumourigenesis during this period, via upregulation of pro-apoptotic factors and potentially through upregulation of *Hsd11b1*, which may protect the breast from low levels of glucocorticoids through their inactivation [70]. We suggest that during periods of stress, levels of unliganded GR are lowered due to a shift towards liganded GR signaling, and it is thus less able to fulfill its protective, pro-apoptotic role. According to this model, downregulation or loss of constitutive activity of unliganded GR would be selected for during cellular transformation since this would confer cells with the ability to resist apoptosis. As reported previously, long-term epigenetic regulation of GR (specifically promoter methylation) represents a mechanism through which an individual’s susceptibility to stress may be altered [71]. Furthermore, low expression of GR has been associated with poorer outcome in estrogen receptor (ER) positive breast cancers [72], and the GR gene *NR3C1* has been reported to be mutated in triple-negative breast cancers, indicating that inactivation of GR is part of the transformation process in these tumours [73]. A reduction in GR levels as a consequence of promoter methylation or mutation would subsequently result in decreased signaling to pro-apoptotic targets due to the loss of positive regulation by unliganded GR, thus potentiating the risk of transformation through the accumulation of abnormal cells. This is consistent with the observed decrease in GR levels in pathologically advanced breast tumours [74]. Thus, we suggest that the activity of unliganded GR in the breast is primarily anti-tumourigenic, and we propose that stress promotes malignant transformation in breast cells since binding of cortisol abolishes the activities of unliganded GR, the result being similar to mutation-induced loss of GR gene expression.

## Conclusion

In conclusion, this study offers additional insight into the role of unliganded GR in the breast, and specifically affords us knowledge of a previously uncharacterized network of transcriptional regulation by unliganded GR. While glucocorticoids and liganded GR appear to suppress apoptosis and facilitate differentiation in the breast, a large proportion of targets of positive regulation by unliganded GR appear to be involved in pro-apoptotic pathways. We suggest that signaling through unliganded GR may represent a mechanism of suppressing the risk of tumourigenesis in the breast by encouraging apoptosis of abnormal cells. Additional study is warranted to further elucidate the role of unliganded GR levels in modulating breast cancer risk.

## Abbreviations

GR: Glucocorticoid receptor; HC: Hydrocortisone; EV: Empty vector; GO: Gene Ontology; GOEAST: Gene Ontology Enrichment Analysis Software Toolkit; IPA: Ingenuity Pathways Analysis.

## Competing interests

The authors declare that they have no competing interests.

## Authors’ contributions

The microarray was completed using services offered by the Queen’s Laboratory for Molecular Pathology. All other experiments, including analysis of the microarray data, were conducted by HDR. HDR participated in the study design and drafted the manuscript. CRM conceived of the study, participated in its design, and edited the manuscript. Both authors read and approved the final manuscript.

## Pre-publication history

The pre-publication history for this paper can be accessed here:

http://www.biomedcentral.com/1471-2407/14/275/prepub

## Supplementary Material

Additional file 1 Primer sequences. Click here for file

Additional file 2 Analyzed microarray data. Click here for file

Additional file 3 Gene Ontology (GO) analyses. Click here for file

Additional file 4 **Transfections with remaining *Ch25h*** **promoter reporters.** Click here for file
