# Supplementary material for: Expression microarray identifies the unliganded glucocorticoid receptor as a regulator of gene expression in mammary epithelial cells
Source: BMC Cancer. 2014 Apr 22;14:275. doi: 10.1186/1471-2407-14-275 (PMC4021255; doi:10.1186/1471-2407-14-275)
Supplement: Additional file 1 — Primer sequences. [file 1471-2407-14-275-S1.pdf]

Additional file 1 – Primer sequences

Table S1: Primers used to construct *Ch25h* promoter fragments

Primer Name	Sequence 5’ to 3’
Ch25h-9-fwd	GGGGATCCCTCGAGCTAGGGCACTCTCCTTGGAT
Ch25h-10-fwd	GGGGATCCCTCGAGGTGTTAAGCCTCCGTTCTCCA
Ch25h-11-fwd	GGGGATCCCTCGAGCACAGGACCCAGTACCAGCA
Ch25h-11.5-fwd	GGGGATCCCTCGAGCCAGGGAATTCTGCTTTGG
Ch25h-12-rev	GGGTCGACTGCTGAGACTATGTCCCACAAT

Table S2: Walking ChIP primers for the *Ch25h* promoter

Primer Name	Sequence 5’ to 3’
Ch25h-1(+)	ACATGTTCTTTTGAGTTGGTATCG
Ch25h-1(-)	TGGAGTGATTTCTCACCTGGA
Ch25h-2(+)	GTGAGAAATCACTCCATATACTT
Ch25h-2(-)	GCCAGACTTTGTAGTCTCCT
Ch25h-3(+)	ACAAAGTCTGGCTGCTTAGGA
Ch25h-3(-)	GACAGCTCATTCATGGTGGGT
Ch25h-4(+)	TGCCTTTGCAATGGGAATCAC
Ch25h-4(-)	ACCAAAGAGCTAGAATGGGCA
Ch25h-5(+)	TCTAGCTCTTTGGTGTAGTCTTGT
Ch25h-5(-)	GAGGTGCACGCTCTTCATTC
Ch25h-6(+)	GCACCTCCTCTAACAGTGAT
Ch25h-6(-)	TGGAATTAAATGCAAATAGTTAAGG
Ch25h-7(+)	TCTTAGTGGTTACCTGTGAAATACA
Ch25h-7(-)	GGATTGTAGCTCTAGGAGCCTG
Ch25h-8(+)	GCTACAATCCCTGTTTTCTTTGG
Ch25h-8(-)	TCCAAGGAGAGTGCCCTAGA
Ch25h-9(+)	CTAGGGCACTCTCCTTGGAT
Ch25h-9(-)	ACCCGTAAAGGCTCACTCG
Ch25h-10(+)	GTGTTAAGCCTCCGTTCTCCA
Ch25h-10(-)	TCCGTTGCAGACAGGGATTG
Ch25h-11(+)	CAACGGACCCAGTACCAGCA
Ch25h-11(-)	ACGTAAAGAACTGTTTGCTTGCC
Ch25h-12(+)	TTTACGTCTGGGAAAGGGCT
Ch25h-12(-)	TGCTGAGACTATGTCCCACAAT
Ch25h-13(+)	CTCAGCATGGGCTGCTACAA
Ch25h-13(-)	TTACGGAAGGCGAGAAGTCC
Ch25h-14(+)	GGACTTCTCGCCTTCCGTAAA
Ch25h-14(-)	GTACAGCCAGGGCACCTTAT
Ch25h-15(+)	TGGCACCTGCTGCACCATAA
Ch25h-15(-)	TCACGTGAAAGGTAAAGATGGTGA

Table S3: Walking ChIP primers for the distal (P1) *Hsd11b1* promoter

Primer Name	Sequence 5' to 3'
Hsd11b1-P1-1(+)	AACCTACAGTTTCTGACCCCT
Hsd11b1-P1-1(-)	ACCTGCTTCCACTTCCAAATCA
Hsd11b1-P1-2(+)	GGCTCAAGAGTTCAAGGTCAGT
Hsd11b1-P1-2(-)	GGCACAGTTGCACACCCATAA
Hsd11b1-P1-3(+)	CTGTGCCTGGCTAACAGTTTAGTTA
Hsd11b1-P1-3(-)	GAGAAGCTGCAAGCAGCAAGG
Hsd11b1-P1-4(+)	CTGCTTGCAGCTTCTCTGGG
Hsd11b1-P1-4(-)	TGCCTGGTGCTGTTTGTATTTTC
Hsd11b1-P1-5(+)	CAAACAGCACCAGGCAATCC
Hsd11b1-P1-5(-)	TGGAAAGTTTCATAAGGCCTATTTT
Hsd11b1-P1-6(+)	ACTGGAGTAGAACCAACAACTAAA
Hsd11b1-P1-6(-)	TTCACTGAGAGGATGGAAACTTTG
Hsd11b1-P1-7(+)	AGTGAAGTCAGGACCCCACAC
Hsd11b1-P1-7(-)	CACATATTATGCACACACATGCAC
Hsd11b1-P1-8(+)	TGTGCATATGTGTGTCTGTGTG
Hsd11b1-P1-8(-)	TCAATACATAATGTGCGCATACAC
Hsd11b1-P1-9(+)	CGCACATTATGTATTGATGTGTGTA
Hsd11b1-P1-9(-)	TGCATACACATGCAGACTCAC
Hsd11b1-P1-10(+)	TGCATGTGTATGCATGTGTGTG
Hsd11b1-P1-10(-)	CTGATACTTTCTGTAGGCCTGTGT
Hsd11b1-P1-11(+)	AGTCTCTCCTTGTGGGGAAGAA
Hsd11b1-P1-11(-)	GCAGTCTGGTGTGGAGATGTTTA
Hsd11b1-P1-12(+)	GTCCCCCTGTCCTTTGGGGAG
Hsd11b1-P1-12(-)	TCTTTGGGGAGGGGCAGGATT
Hsd11b1-P1-13(+)	CCCCTCCCCAAAGAGTGAGA
Hsd11b1-P1-13(-)	GACCTGTTAGCCCTACTGTGT
Hsd11b1-P1-14(+)	AGGGCTAACAGGTCTGAAAAGAA
Hsd11b1-P1-14(-)	GACTTCCCACAGCCTTAGCA

Table S4: Walking ChIP primers for the proximal (P2) *Hsd11b1* promoter

Primer Name	Sequence 5' to 3'
Hsd11b1-P2-1(+)	ATGTACAAGGATCCCCAGAGC
Hsd11b1-P2-1(-)	GTGAGCTTGTGTGTGAGAGC
Hsd11b1-P2-2(+)	GAGGCTCTCACACACAAGCTC
Hsd11b1-P2-2(-)	AAAGTCTTGCTATGCAACCCA
Hsd11b1-P2-3(+)	GGGTTGCATAGCAAGACTTTGA
Hsd11b1-P2-3(-)	GTGACAGTCCAACACTGTCTCT
Hsd11b1-P2-4(+)	ACTTGAGCCACTGATCAGCAT
Hsd11b1-P2-4(-)	AAGAAAGGGATGAGAGAGTGGG
Hsd11b1-P2-5(+)	ATCCCTTTCTTTTCTCTCTGTCTTT
Hsd11b1-P2-5(-)	GGACTCCACGTTCCTTCACG
Hsd11b1-P2-6(+)	TCCTGTCTGTCATGTGTCTTGG
Hsd11b1-P2-6(-)	GCCCAGAGGAGACGAAACAAT

Hsd11b1-P2-7(+)	CGTCTCCTCTGGGCCTTAAAT
Hsd11b1-P2-7(-)	TGTGAGTAACTGCAGAGCATGT
Hsd11b1-P2-8(+)	CTCACAGTTCAGGATTGGCTTT
Hsd11b1-P2-8(-)	ATCCAACAGGAGCATCAAGACA
Hsd11b1-P2-9(+)	TTACCTGGGACTTGGATGCTC
Hsd11b1-P2-9(-)	CTATAGCCACACTGCTCGCTC
Hsd11b1-P2-10(+)	TGGTCTAGATCTTCTAGTCTGGC
Hsd11b1-P2-10(-)	CACTCCAGTTCAATGAAAAAGCAT
Hsd11b1-P2-11(+)	GAACTGGAGTGTGGGGCTTAT
Hsd11b1-P2-11(-)	GCACCTAAGAACCCCTGAGC
Hsd11b1-P2-12(+)	AGGTGCTGTTTCTCTCTGTGT
Hsd11b1-P2-12(-)	AGAGGCAACTTCCAGATGGC
Hsd11b1-P2-13(+)	TCTGGAAGTTGCCTCTTACTCA
Hsd11b1-P2-13(-)	AGAGCTTTCTACAACCTCCCA
Hsd11b1-P2-14(+)	GCAGGTTTTCTTCGTGTGTCC
Hsd11b1-P2-14(-)	GGGTTTGAATCAGTGATGTAGGG
