# Supplementary material for: Expression microarray identifies the unliganded glucocorticoid receptor as a regulator of gene expression in mammary epithelial cells
Source: BMC Cancer. 2014 Apr 22;14:275. doi: 10.1186/1471-2407-14-275 (PMC4021255; doi:10.1186/1471-2407-14-275)
Supplement: Additional file 3 — Gene Ontology (GO) analyses. [file 1471-2407-14-275-S3.pdf]

Additional file 3 – Gene Ontology (GO) analyses

Gene Ontology

In order to analyze potential functional trends in our microarray data, we performed a functional analysis of the lists of differentially expressed up and downregulated genes using GOEAST (Gene Ontology Enrichment Analysis Software Toolkit) [24]. This program enabled the determination of the most highly represented gene ontology (GO) categories in response to GR depletion, and the number of genes in each set (up and downregulated) belonging to those categories. This analysis revealed that a large number of the 260 upregulated genes (*ie*. genes negatively regulated by unliganded GR) were involved in various developmental and morphogenetic processes (total >200 genes; false discovery rate (FDR) < 1%), as well as "protein binding" (>50 genes; FDR < 1%) (Figure A1). The most abundantly enriched categories for the 343 downregulated genes (*ie*. genes positively regulated by unliganded GR) were "binding" (>120 genes; FDR < 1%), "cytoplasm" (>80 genes; FDR < 1%), and "metabolic processes" (>80 genes; FDR < 1%), as well as various processes related to immune system regulation and signaling (total >300 genes; FDR < 1%) (Figure A2). Given the known role of GR in the regulation of inflammation and the innate immune response, loss of its expression was expected to downregulate certain factors in these processes. It was of note that virtually no overlap of GO categories existed between the up and downregulated sets of genes. Furthermore, when our gene lists were analyzed for factors involved in apoptotic processes, it was of note that several targets of positive regulation by unliganded GR (*ie*. downregulated in shGR-19 cells) were pro-apoptotic, while a number of genes negatively regulated by unliganded GR (*ie*. upregulated in shGR-19 cells) appeared to be anti-apoptotic.

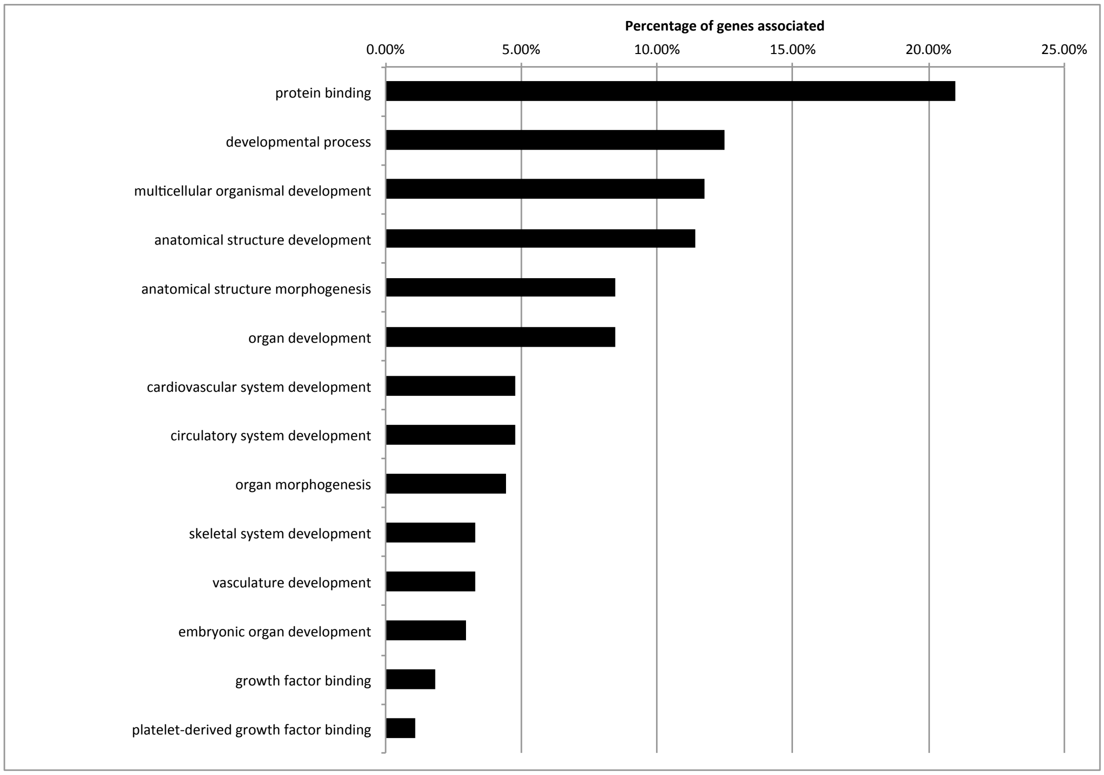

Figure S1: Gene Ontology (GO) functional analysis of targets of negative regulation by unliganded GR.

Statistically enriched Gene Ontology terms of targets of negative regulation by unliganded GR (*ie*. genes upregulated in shGR-19 cells relative to EV-50 control cells) as determined by the Gene Ontology Enrichment Analysis Software Toolkit (GOEAST). Numbers represent the percentages of genes identified associated with a GO biological process, cellular component or molecular function.

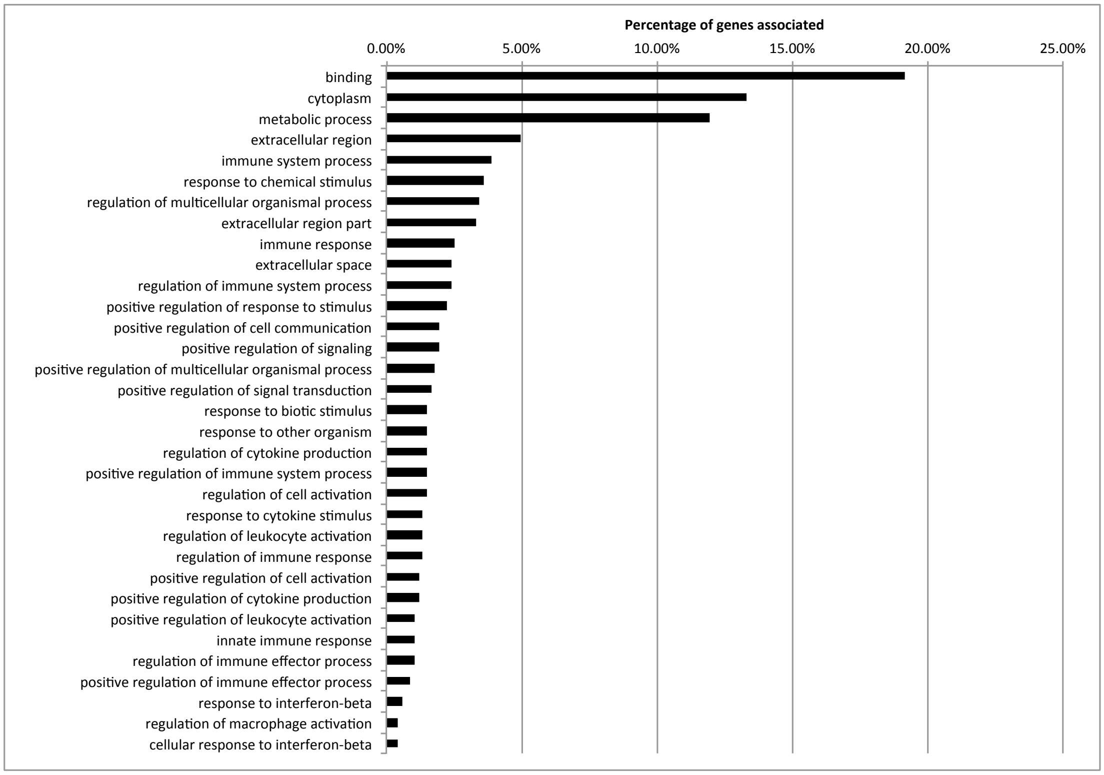

Figure S2: Gene Ontology (GO) functional analysis of targets of positive regulation by unliganded GR.

Statistically enriched Gene Ontology terms of targets of positive regulation by unliganded GR (*ie*. genes downregulated in shGR-19 cells relative to EV-50 control cells) as determined by the Gene Ontology Enrichment Analysis Software Toolkit (GOEAST). Numbers represent the percentages of genes identified associated with a GO biological process, cellular component or molecular function.
