# Supplementary material for: Expression microarray identifies the unliganded glucocorticoid receptor as a regulator of gene expression in mammary epithelial cells
Source: BMC Cancer. 2014 Apr 22;14:275. doi: 10.1186/1471-2407-14-275 (PMC4021255; doi:10.1186/1471-2407-14-275)
Supplement: Additional file 4 — Transfections with remaining Ch25h promoter reporters. [file 1471-2407-14-275-S4.pdf]

Additional file 4 – Transfections with remaining *Ch25h* promoter reporters

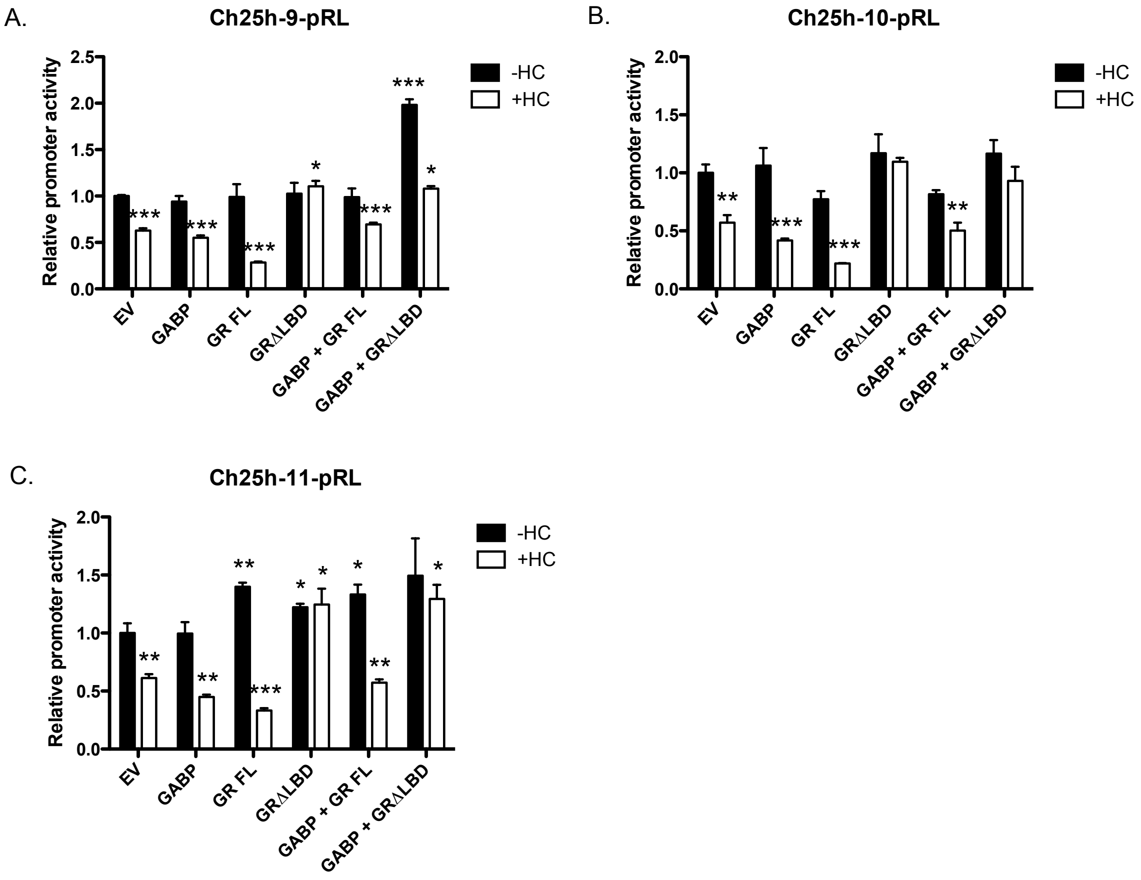

Figure S3: GRΔLBD activates the *Ch25h* promoter in the presence and absence of HC. EPH-4 cells were transiently transfected with the *Ch25h* promoter reporters **A.** Ch25h-9-pRL, **B.** Ch25h-10-pRL and **C.** Ch25h-11-pRL as well as with expression vectors for GABPα/β (GABP), full-length GR (GR FL) and GR lacking the ligand binding domain (GRΔLBD). Cells were treated 24 hours after transfection with either ethanol vehicle (-HC) or 1 μg/mL HC (+HC) and assayed for luciferase activity following a 48 hour incubation. Bars represent the mean of technical replicates, and error bars represent standard deviation (N = 3). Statistically significant changes in *Ch25h* promoter activity relative to the EV (-HC) transfection are indicated: one asterisk, $p<0.05$ (significant); two asterisks, $p<0.005$ (very significant); three asterisks, $p<0.0005$ (very highly significant).
